# Genetic susceptibility to aminoglycoside cochleotoxicity in South African multidrug-resistant and rifampicin-resistant tuberculosis patients

**DOI:** 10.4102/sajcd.v73i1.1180

**Published:** 2026-08-31

**Authors:** Nazanin Ghafari, Lebogang Ramma, Richard Court, Noluthando Manyisa, Helen McIlleron, Lucretia Petersen

**Affiliations:** 1 Department of Health and Rehabilitation Sciences, Faculty of Health Sciences, University of Cape Town, Cape Town, South Africa; 2 Division of Clinical Pharmacology, Department of Medicine, Faculty of Health Sciences, University of Cape Town, Cape Town, South Africa; 3 Division of Human Genetics, Department of Pathology, Faculty of Health Sciences, University of Cape Town, Cape Town, South Africa

**Keywords:** multidrug-resistant tuberculosis, rifampicin-resistant tuberculosis, kanamycin, aminoglycosides, cochleotoxicity, ototoxicity, mitochondrial variants, ultra-high-frequency audiometry

## Abstract

**Background:**

South Africa has a high burden of multidrug-resistant tuberculosis (MDR-TB) and rifampicin-resistant tuberculosis (RR-TB). At the time of this study, kanamycin formed part of the South African MDR/RR-TB treatment regimen. Although treatment has shifted towards shorter all-oral regimens, aminoglycoside-related ototoxicity remains clinically relevant.

**Objectives:**

This study investigated the association between two mitochondrial variants, m.15312T>C (I189T in *MT-CYB [mitochondrially encoded cytochrome b]*) and m.10114T>C (I19T in *MT-ND3 [mitochondrially encoded NADH]*), and susceptibility to cochleotoxicity in South African patients receiving kanamycin-based MDR/RR-TB treatment.

**Method:**

A prospective cohort study was conducted in Cape Town, South Africa. Hearing thresholds from 0.25 kHz to 16 kHz were monitored at baseline and at 4 weeks, 8 weeks and 12 weeks after treatment initiation. Cochleotoxicity was defined using the American Speech-Language-Hearing Association’s significant threshold shift criteria. Mitochondrial variants were identified using polymerase chain reaction and Sanger sequencing. Fisher’s exact tests were used to explore associations between variants and cochleotoxicity.

**Results:**

Hearing data were analysed for 102 participants. Cochleotoxicity developed in 84 participants (82.4%). The m.15312T>C variant was detected in three of 78 successfully sequenced participants, and m.10114T>C in four of 80. All variant carriers had cochleotoxicity. Statistically significant associations were not demonstrated for m.15312T>C (*p* = 1.000) or m.10114T>C (*p* = 1.000).

**Conclusion:**

The variants were observed only among participants who developed cochleotoxicity, but no statistically significant associations were observed. The findings are exploratory and require validation before clinical screening.

**Contribution:**

This study supports future genetic-susceptibility research in African aminoglycoside-exposed populations.

## Introduction

South Africa remains a high-burden country for tuberculosis, including drug-resistant tuberculosis, and multidrug-resistant tuberculosis/rifampicin-resistant tuberculosis (MDR/RR-TB) continues to represent an important public-health and treatment-monitoring concern nationally (World Health Organization [WHO], 2024). At the time of recruitment, participants in this cohort were receiving kanamycin-based MDR/RR-TB treatment in accordance with the South African national guidance in use at the time (South African Department of Health, 2013). Since then, MDR/RR-TB treatment policy has shifted substantially towards shorter, all-oral, and injectable-sparing regimens, including newer bedaquiline-, pretomanid-, and linezolid-containing regimens for eligible patients (South African National Department of Health, 2023; WHO, 2022). However, amikacin may still be included in longer MDR/RR-TB regimens for adults when drug susceptibility has been demonstrated, and adequate monitoring for adverse reactions can be ensured (WHO, 2022). This policy shift reduces the routine use of injectable aminoglycosides, but it does not remove the scientific and clinical relevance of aminoglycoside-related cochleotoxicity. Kanamycin and amikacin are structurally related aminoglycosides; amikacin is a semisynthetic analogue or derivative of kanamycin (Ramirez & Tolmasky, 2017; Ristuccia & Cunha, 1985). Both agents have recognised cochleotoxic potential, and aminoglycoside-related ototoxicity remains an important clinical concern (Jiang et al., 2017; Petersen & Rogers, 2015). Therefore, historical cohorts exposed to kanamycin remain important for understanding mechanisms of susceptibility and for informing future risk-stratification research, particularly in settings where aminoglycoside exposure occurs or injectable agents are considered for selected patients with limited treatment options, drug intolerance, resistance patterns or regimen constraints.

Aminoglycoside-induced hearing loss is multifactorial, with genetic predisposition recognised as an important contributor to inter-individual susceptibility. Mitochondrial variants associated with aminoglycoside ototoxicity have been reported across populations, although their prevalence and clinical significance may vary by population group (Gao et al., 2017; Konings et al., 2008; Lévêque et al., 2007; Li et al., 2004; Lu et al., 2010). Given South Africa’s high genetic diversity, local investigations are important for identifying population-specific susceptibility markers (Choudhury et al., 2018; Krause, 2015). A previous South African study identified the m.1555A>G mutation in a family with streptomycin-induced deafness, supporting the relevance of mitochondrial susceptibility to aminoglycoside-related hearing loss in this setting (Gardner et al., 1997). The rationale for selecting m.15312T>C in *MT-CYB* (mitochondrially encoded cytochrome b) and m.10114T>C in *MT-ND3* mitochondrially encoded NADH) was based on Human’s (2009) South African study, which identified these variants as candidate susceptibility markers in the context of aminoglycoside-associated hearing loss. However, evidence for these variants remains limited, and replication in independent cohorts is required. Therefore, this study did not aim to confirm pathogenicity but rather to explore whether these two candidate mitochondrial variants were associated with cochleotoxicity among South African MDR/RR-TB patients exposed to kanamycin.

## Research methods and design

This prospective cohort study investigated the association between susceptibility to cochleotoxicity and two candidate mitochondrial variants, m.15312T>C (I189T in *MT-CYB*) and m.10114T>C (I19T in *MT-ND3*), in patients with MDR/RR-TB in Cape Town, South Africa. The study was conducted at Brooklyn Chest Hospital and the DP Marais Hospital, both of which were referral centres for MDR/RR-TB management.

### Study design and sampling

This prospective cohort study used convenience sampling. Eligible participants were adults with MDR/RR-TB who were initiating kanamycin-based treatment at the participating hospitals. Patients with middle-ear pathology were excluded from the study.

The sample-size estimate was based on detecting a clinically relevant 10 dB difference in pure-tone audiometric thresholds between baseline and follow-up assessments, using American Speech-Language-Hearing Association (ASHA) Significant Threshold Shift (STS) criteria and assuming a common standard deviation of 10 dB. Based on this calculation, a sample size of 60 participants would provide 99% power using a two-sided test at the 5% significance level. However, this calculation was based on changes in audiological threshold rather than on detecting associations between rare mitochondrial variants and cochleotoxicity. Because no prior frequency estimates were available for m.15312T>C or m.10114T>C in this South African MDR/RR-TB population, the study was not formally powered for genetic association testing. This limitation is acknowledged in the interpretation of the genetic findings.

At the time of the study, the standard MDR/RR-TB regimen consisted of pyrazinamide, moxifloxacin, kanamycin, terizidone, and either ethionamide or isoniazid. Ethambutol was included according to local guidelines when resistance was considered low. Participants received kanamycin daily, six times per week, at 15 mg/kg per dose according to South African Department of Health guidelines. Kanamycin dosage was adjusted for renal dysfunction at the discretion of the treating clinician. Renal function was assessed at 4 weeks, 8 weeks and 12 weeks after treatment initiation using the Cockcroft-Gault method to calculate creatinine clearance.

### Audiological monitoring

Audiological monitoring was performed at baseline and at approximately 4 weeks, 8 weeks and 12 weeks after treatment initiation. The assessment included case history, otoscopic examination, tympanometry and pure-tone audiometry, including ultra-high-frequency audiometry up to 16 kHz. Pure-tone thresholds were measured across conventional frequencies and ultra-high frequencies. For reporting the distribution of threshold shifts, frequencies were grouped into low conventional frequencies, 0.25 kHz – 2 kHz; high conventional frequencies, 3 kHz – 8 kHz; and ultra-high frequencies, 9 kHz – 16 kHz.

Cochleotoxicity was determined by comparing each follow-up audiogram with the participant’s baseline audiogram using ASHA STS criteria (ASHA, 1994). Cochleotoxicity was defined as a 20 dB decrease at any one test frequency, a 10 dB decrease at any two adjacent frequencies or loss of response at three consecutive frequencies where responses were previously obtained. Participants required a minimum of two valid audiograms to be included in the hearing analysis. Results were shared with the patient, resident audiologist and medical doctor for appropriate management or treatment modification.

### Genetic sampling and sequencing

Blood samples were collected for genetic analysis, immediately centrifuged, and the buffy coat was stored at −70 °C. Deoxyribonucleic acid (DNA) was extracted using the chemagic 360 instrument according to the manufacturer’s instructions. Deoxyribonucleic acid quality and quantity were assessed using the Nanodrop ND100 spectrophotometer (Nanodrop Technologies, Wilmington, DE, United States [US]) and DNA integrity was confirmed using a 1% agarose gel.

Direct cycle sequencing was performed using custom primers for *MT-CYB* gene (mitochondrially encoded cytochrome B) and *MT-ND3* gene (mitochondrially encoded NADH: ubiquinone oxidoreductase core subunit 3) (Table 1). Primer specificity was evaluated using Primer-Basic Local Alignment Search Tool (BLAST) (Ye et al., 2012) and in silico polymerase chain reaction (PCR). Primer annealing was performed at 58.5 °C for *MT-CYB* and 54 °C for *MT-ND3* for 30 s. Sequencing of PCR products was performed using the BigDye Terminator v3.1 Cycle Sequencing Kit (Thermo Fisher Scientific, Applied Biosystems, Waltham, MA, US) and an ABI 3130XL Genetic Analyzer (Applied Biosystems and Hitachi, Tokyo, Japan).

**TABLE 1:** Primer sequences used for amplification of target regions of *MT-ND3* and *MT-CYB*.

Target region	Forward/reverse	Primer sequence (5′–3′)	Expected product size
*MT-ND3*	Forward	AGT ACT TCG AGT CTC CCT TC	881 bp
	Reverse	GTG GGT GTT GAG GGT TAT G	
*MT-CYB*	Forward	CTT ACT ATC CGC CAT CCC ATA C	1141 bp
	Reverse	CTG CGG CTA GGA GTC AAT AAA	

bp, base pairs; *MT-ND3*, mitochondrially encoded NADH:ubiquinone oxidoreductase core subunit 3; *MT-CYB*, mitochondrially encoded cytochrome b.

### Data handling and statistical analysis

Data were captured in Microsoft Excel and double-checked for accuracy before analysis. Descriptive statistics were used to summarise demographic and clinical characteristics, hearing outcomes, and variant frequencies. The minor allele frequency was calculated for both target variants.

Fisher’s exact tests were used to explore associations between each target variant and cochleotoxicity because of low expected cell counts. Results were interpreted cautiously because the variants were rare and the number of participants without cochleotoxicity was small. No multivariable model was fitted because the number of variant carriers was insufficient to support adjusted analysis. Therefore, potential contributors to cochleotoxicity, including human immunodeficiency virus (HIV) status, renal function, previous MDR/RR-TB treatment, baseline hearing status and treatment exposure, could not be controlled for in an adjusted model.

### Ethical considerations

Ethical clearance to conduct this study was obtained from the University of Cape Town Human Research Ethics Committee (No. HREC 065/2015 and HREC 595/2018). Written informed consent was obtained from each participant in their preferred language (English, Afrikaans, or isiXhosa). Consent for publication of anonymised data was also obtained.

## Results

A total of 147 participants were initially recruited. Data from 45 participants (30.6%) were excluded because they did not complete the minimum of two valid hearing assessments required for analysis. Reasons included early discharge, withdrawal, death before follow-up, or being too ill to complete assessments. The final analytic sample included 102 participants. Baseline demographic and clinical characteristics are summarised in Table 2.

**TABLE 2:** Baseline demographic and clinical characteristics of study participants (*N* = 102).

Variable	Value		Median	Range	IQR
	*n*	%			
Male	58	56.9	-	-	-
HIV infected	65	63.7	-	-	-
With previous MDR-TB treatment	24	23.5	-	-	-
Age (years)	-	-	34.9	27.2–42.2	-
BMI (kg/m^2^)	-	-	17.3	-	15.6–18.9
Creatinine clearance, mL/min	-	-	79.7	-	58.8–98.8

IQR, interquartile range; HIV, human immunodeficiency virus; MDR-TB, multidrug-resistant tuberculosis; BMI, body mass index.

### Hearing outcomes

Of the 102 participants with analysable hearing data, 84 participants (82.4%) demonstrated a STS on their final available audiogram according to the ASHA cochleotoxicity criteria (ASHA, 1994). Among these 84 participants, 61 participants (73%) demonstrated bilateral STS, and 17 participants (20%) demonstrated unilateral STS. A further six participants (7%) demonstrated unilateral STS, while the opposite ear had middle-ear pathology and was excluded from analysis.

The frequency range affected by STS changed over the 12-week monitoring period (Table 3). At 4 weeks, 58 participants (57%) demonstrated STS, with 43 participants (42%) showing STS in the ultra-high-frequency range. By 8 weeks, 72 participants (71%) demonstrated STS; by 12 weeks, 84 participants (82%) demonstrated STS. The number of participants classified as having STS limited to the ultra-high-frequency range decreased over time, while conventional frequency involvement increased from 15 participants (15%) at 4 weeks to 33 participants (33%) at 8 weeks and 48 participants (47%) at 12 weeks.

**TABLE 3:** Distribution of significant threshold shift by frequency range and treatment duration among participants with analysable hearing data (*N* = 102).

Frequency range/timing	Ultra-high frequency 9 kHz–16 kHz		High conventional 3 kHz–8 kHz		Low conventional 0.25 kHz–2 kHz		Total with STS 0.25 kHz–16 kHz	
	*n*	%	*n*	%	*n*	%	*n*	%
Participants with STS during 0–4 weeks	43	42	6	6	9	9	58	57
Participants with STS during 0–8 weeks	39	38	15	15	18	18	72	71
Participants with STS during 0–12 weeks	36	35	27	26	21	21	84	82

Note: Percentages were calculated using the full analysable sample, *N* = 102. Each participant was classified once at each time point according to the lowest frequency range affected by STS in the most severely affected ear. A reduction in participants classified in the ultra-high frequency-only category over time reflects progression into conventional frequency involvement rather than recovery.

STS, significant threshold shift.

The degree of hearing impairment in the conventional frequency range, 0.25 kHz – 8 kHz, was classified according to the WHO grading system (WHO, 2008) using the most severely affected ear (Table 4). Among the 84 participants with STS on their last audiogram, 47 participants (56%) had no conventional-frequency hearing impairment, while 37 participants (44%) had slight to severe hearing impairment. All 37 participants with conventional-frequency impairment had sensorineural hearing loss.

**TABLE 4:** Degree of conventional-frequency hearing impairment among participants with significant threshold shift on the last audiogram (*n* = 84).

Degree	No impairment ≤ 25 dB	Slight 26 dB–40 dB	Moderate 41 dB–60 dB	Severe 61 dB–80 dB	Profound ≥ 81 dB
Number of participants	47	17	10	10	0
Percentage (%)	56	20	12	12	0

Note: Degree of hearing impairment was classified according to the World Health Organization (WHO) grading system (WHO, 2008) using the most severely affected ear. Conventional frequencies were defined as 0.25 kHz – 8 kHz.

Cochleotoxicity severity was further described using Common Terminology Criteria for Adverse Events version 5 (CTCAEv5), TUNE ototoxicity grading system, and University of Cape Town (UCT) criteria (National Cancer Institute, 2017; Ramma, 2016; Theunissen et al., 2014) (Table 5). Common Terminology Criteria for Adverse Events version 5 and TUNE were used as established ototoxicity grading systems, while the UCT criteria were used as a locally used clinical/research grading approach in this setting. The proportion of participants classified with grade greater than 0 varied across grading systems: 45 participants (53.5%) using CTCAEv5, 65 participants (77%) using TUNE and 84 participants (100%) using UCT criteria. These results are presented descriptively to show how cochleotoxicity classification varied according to the grading system applied.

**TABLE 5:** Cochleotoxicity grading among participants with significant threshold shift (*n* = 84).

Grade	CTCAEv5		TUNE		UCT	
	*n*	%	*n*	%	*n*	%
0	39	46.5	19	23	0	0
1/1a	16	19	16	19	10	12
1b	-	-	13	15.5	37	44
2/2a	13	15.5	22	26	17	20
2b	-	-	7	8.3	10	12
3	16	19	7	8.3	10	12
4	-	-	0	0	0	0

Total with grade > 0	45	53.5	65	77	84	100

Note: CTCAEv5 and TUNE were applied as established ototoxicity grading systems. University of Cape Town criteria were applied as a locally used clinical/research grading approach in this setting, based on Ramma (2016), and should be interpreted as contextual descriptive information. Grades were assigned using the most severely affected ear. Dashes indicate grading categories not used in the relevant grading scale.

CTCAEv5, Common Terminology Criteria for Adverse Events version 5; UCT, University of Cape Town.

### Genetic findings and statistical analysis

**FIGURE 1:**
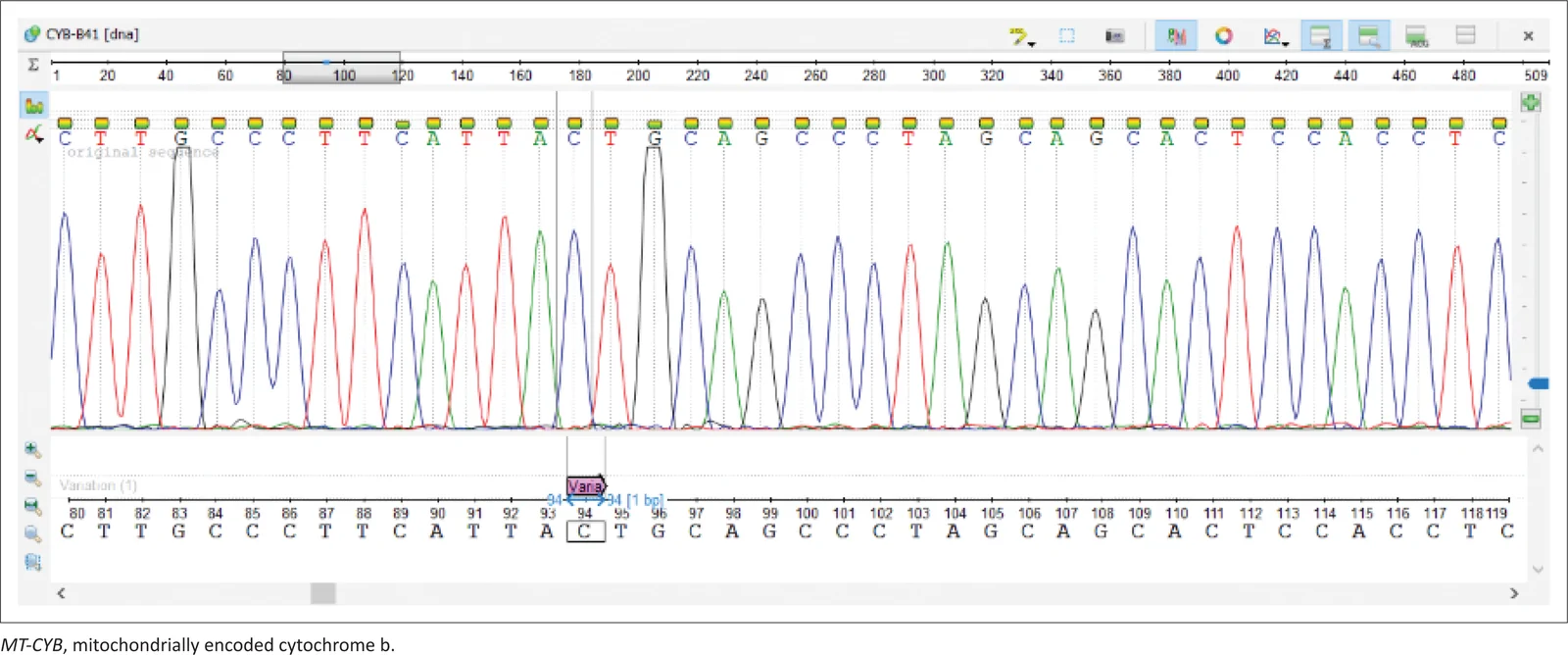
Chromatogram showing the homozygous m.15312T>C target variant in mitochondrially encoded cytochrome b. The position of the variant is indicated with the purple arrow.

The m.15312T>C variant in *MT-CYB* was detected in three of 78 successfully sequenced participants (Table 6; Figure 1). All three participants developed cochleotoxicity. The variant was not detected among participants without cochleotoxicity. Fisher’s exact test did not demonstrate a statistically significant association between m.15312T>C and cochleotoxicity, 3 of 66 versus 0 of 12, *p* = 1.000.

**TABLE 6:** Prevalence of m.15312T>C (I189T in *MT-CYB*) and m.10114T>C (I19T in *MT-ND3*) variants in participants with successfully sequenced deoxyribonucleic acid samples (*n* = 78 for *MT-CYB* gene; *n* = 80 for *MT-ND3*).

Gene	Genotype	SNP	Variant	With STS		Without STS		MAF
				*n*	%	*n*	%	
*MT-CYB*	Homozygous	rs1603335215	m.15312T>C	3	4.54	0	0	0.045
*MT-ND3*	Homozygous	rs779734442	m.10114T>C	3	5.35	0	0	0.046
	Heterozygous	rs779734442	m.10114T>C	1	1.53	0	0	0.015

Note: For *MT-CYB*, with STS n = 66 and without STS n = 12. For *MT-ND3*, with STS *n* = 65 and without STS *n* = 15.

STS, significant threshold shift; SNP, single nucleotide polymorphism; MAF, minor allele frequency; *MT-CYB*, mitochondrially encoded cytochrome b; *MT-ND3*, mitochondrially encoded NADH:ubiquinone oxidoreductase core subunit 3.

**FIGURE 2:**
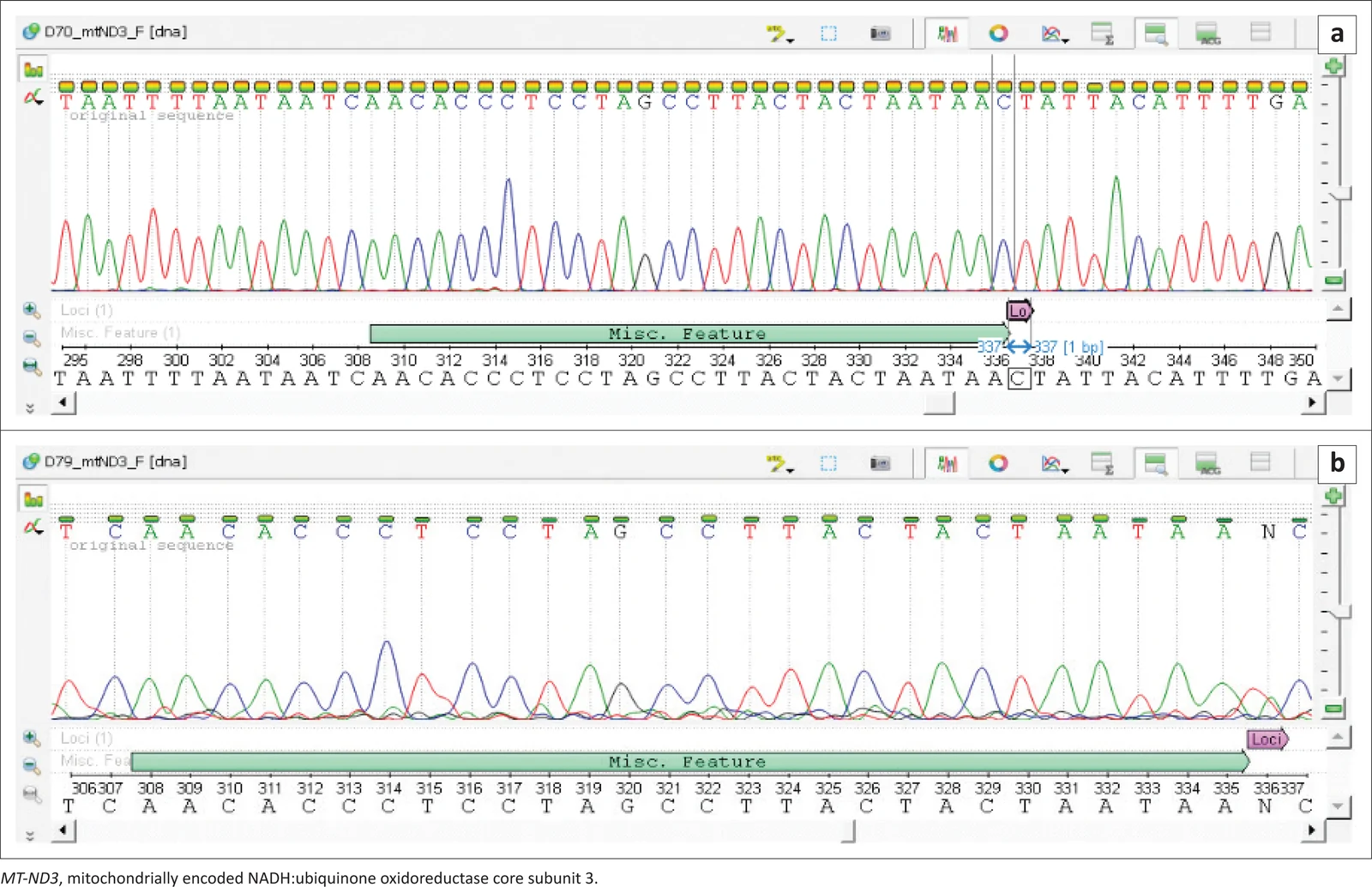
Chromatogram showing the m.10114T>C target variant in mitochondrially encoded NADH:ubiquinone oxidoreductase core subunit 3. The position of the variant is indicated with the purple arrow: (a) homozygous variant and (b) heterozygous variant.

The m.10114T>C variant in *MT-ND3* was detected in 4 of 80 successfully sequenced participants (Table 6; Figure 2). All four participants developed cochleotoxicity. The variant was not detected among participants without cochleotoxicity. Fisher’s exact test did not demonstrate a statistically significant association between m.10114T>C and cochleotoxicity, 4 of 65 versus 0 of 15, *p* = 1.000.

Non-target variants are summarised in Table 7. Two participants carried the m.15301G>A variant near the m.15312T>C site, and seven participants carried the m.10115T>C variant near the m.10114T>C site.

**TABLE 7:** Prevalence of non-target variants in participants with successfully sequenced deoxyribonucleic acid samples.

Gene	Genotype	SNP	Variant	With STS		Without STS		MAF
				*n*	%	*n*	%	
*MT-CYB*	Homozygous	rs193302991	m.15301G>A	2	3.03	0	0	0.030
*MT-ND3*	Homozygous	rs38999188	m.10115T>C	5	7.69	2	13.33	0.092

Note: For *MT-CYB*, with STS n = 66 and without STS *n* = 12. For *MT-ND3*, with STS *n* = 65 and without STS *n* = 15, *n* = 78 for mitochondrially encoded cytochrome b gene; *n* = 80 for mitochondrially encoded NADH:ubiquinone oxidoreductase core subunit 3.

STS, significant threshold shift; SNP, single nucleotide polymorphism; MAF, minor allele frequency; *MT-CYB*, mitochondrially encoded cytochrome b gene; *MT-ND3*, mitochondrially encoded NADH:ubiquinone oxidoreductase core subunit 3.

These results show that both target variants were observed only in participants who developed cochleotoxicity, but statistical evidence of association was not demonstrated in this cohort.

## Discussion

### Interpretation of cochleotoxicity findings

The high incidence of cochleotoxicity in this cohort is consistent with the established ototoxic risk of injectable aminoglycosides and may also reflect the sensitivity of ultra-high frequency audiometry (Jiang et al., 2017; Petersen & Rogers, 2015). The 82.4% incidence observed in this study is higher than the 57% high frequency hearing loss reported by Harris et al. (2012) among South African MDR-TB patients treated with aminoglycosides, and higher than the 48% sensorineural or mixed hearing loss reported by Ghafari et al. (2015) among children with tuberculosis receiving ototoxic medication. This difference may partly reflect this study’s use of ASHA STS criteria and ultra-high frequency audiometry up to 16 kHz, which are sensitive to early cochlear changes. The expanded hearing results showed that STS was detected earliest in the ultra-high frequency range and later extended into conventional frequencies in a substantial proportion of participants. This pattern supports the value of ultra-high frequency audiometry for early ototoxicity monitoring, as conventional frequency impairment may occur only after earlier ultra-high frequency changes (Konrad-Martin et al., 2005).

The hearing results also show the importance of distinguishing between STS and clinically graded conventional-frequency hearing impairment. Although 84 participants met ASHA criteria for STS, 47 did not yet meet criteria for conventional frequency hearing impairment (ASHA, 1994; WHO, 2008). This suggests that ASHA-based monitoring and ultra-high-frequency audiometry may identify early cochlear changes before they are reflected in conventional hearing-impairment categories.

Cochleotoxicity severity varied according to the grading system applied. Common Terminology Criteria for Adverse Events version 5 classified fewer participants as having grade greater than 0 compared with TUNE and UCT criteria (National Cancer Institute, 2017; Ramma, 2016; Theunissen et al., 2014). This variation indicates that cochleotoxicity prevalence and severity estimates are influenced by the sensitivity and classification structure of the grading scale used. In this cohort, the UCT criteria classified the highest proportion of participants as having cochleotoxicity; however, because these criteria are locally applied and are not as widely standardised internationally as CTCAEv5 or TUNE, UCT-based classifications should be interpreted cautiously and as contextual descriptive information.

### Interpretation of genetic findings

The target variants m.15312T>C in *MT-CYB* and m.10114T>C in *MT-ND3* were detected only among participants who developed cochleotoxicity. This observation is consistent with the possibility that these variants may represent candidate susceptibility markers, as previously suggested in South African work on aminoglycoside-associated hearing loss (Human, 2009). However, the findings should not be interpreted as confirmation of pathogenicity. The number of variant carriers was small, and Fisher’s exact tests did not demonstrate statistically significant associations for either variant. The results are therefore exploratory and hypothesis-generating and require validation in larger cohorts with broader genetic panels.

The absence of statistically significant associations is important for interpretation. Although the variants were observed only in participants with cochleotoxicity, the high overall incidence of cochleotoxicity and the small number of participants without cochleotoxicity reduced the ability to distinguish genetic susceptibility from the broader ototoxic effect of kanamycin exposure. Therefore, the findings should be viewed as preliminary evidence supporting further investigation rather than evidence for clinical implementation.

### Current clinical relevance

This study was conducted in the context of kanamycin-based MDR/RR-TB treatment in use at the time of recruitment, in accordance with South African guidance then in force (South African Department of Health, 2013). Current treatment recommendations have shifted towards shorter all-oral and injectable-sparing regimens, reducing the routine use of injectable aminoglycosides (South African National Department of Health, 2023; WHO, 2022). However, this does not eliminate the relevance of aminoglycoside-related ototoxicity research. Amikacin, a structurally related aminoglycoside with recognised cochleotoxic potential, may still be considered in selected MDR/RR-TB patients when susceptibility is demonstrated and adequate monitoring is available (Ramirez & Tolmasky, 2017; Ristuccia & Cunha, 1985; WHO, 2022). Therefore, evidence from historical kanamycin-exposed cohorts remains relevant for understanding aminoglycoside cochleotoxicity and potential susceptibility mechanisms.

The study also contributes to the limited evidence base on mitochondrial variation and aminoglycoside-induced hearing loss in African populations. Given the genetic diversity of South African populations and the limited availability of local genetic susceptibility data, exploratory studies such as this can help identify candidate variants for future validation (Choudhury et al., 2018; Krause, 2015).

### Strengths and limitations

This study has several strengths. Firstly, the prospective cohort design enabled monitoring of hearing thresholds from baseline through follow-up, rather than relying on retrospective auditory status data. Secondly, cochleotoxicity was determined by comparing each participant’s follow-up audiogram with their own baseline audiogram using ASHA STS criteria (ASHA, 1994). Thirdly, the inclusion of ultra-high frequency audiometry up to 16 kHz increased sensitivity to early cochlear changes before progression into the conventional frequency range (Konrad-Martin et al., 2005). Fourthly, the study contributes local evidence from a South African MDR/RR-TB cohort, addressing limited data on mitochondrial variation and aminoglycoside-induced cochleotoxicity in African populations.

This study also has limitations. Convenience sampling may have introduced selection bias and may limit generalisability. Attrition was high, with 45 of 147 recruited participants excluded because they did not complete the minimum number of valid hearing assessments. Participants who died, withdrew, were discharged early or were too ill to complete follow-up may have differed clinically from those retained in the analysis, introducing possible attrition bias.

The sample size calculation was based on detecting a clinically relevant 10 dB difference in pure-tone audiometric thresholds between baseline and follow-up assessments. Therefore, the study was designed to detect changes in audiological thresholds, but it was not powered for rare-variant genetic association analysis. Deoxyribonucleic acid was unavailable, or sequencing was unsuccessful for a proportion of participants, further reducing the sample size for genetic analysis. The low number of variant carriers limited statistical power and prevented adjusted modelling.

Known aminoglycoside-associated mitochondrial variants, including m.1555A>G and m.1494C>T, were not screened. This limits the interpretation of the independent contribution of m.15312T>C and m.10114T>C, because established mitochondrial variants have previously been associated with aminoglycoside-induced hearing loss (Gao et al., 2017; Lu et al., 2010). Potential confounders, including HIV status, renal function, previous MDR/RR-TB treatment, baseline hearing status, cumulative aminoglycoside exposure, and other ototoxic exposures, could not be fully controlled. If the UCT grading criteria are retained, UCT-based classifications should also be interpreted cautiously because these criteria are locally applied and are not as widely standardised internationally as CTCAEv5 or TUNE.

These limitations mean that the genetic findings should be interpreted cautiously as exploratory and hypothesis-generating.

### Implications and recommendations

Future studies should use larger, adequately powered cohorts and broader genetic panels that include established aminoglycoside associated variants, such as m.1555A>G and m.1494C>T, as well as candidate variants identified in African populations. Where possible, future analyses should adjust for relevant clinical risk factors, including HIV status, renal function, prior MDR/RR-TB treatment, baseline hearing status, cumulative aminoglycoside exposure, and concurrent ototoxic medications.

The present findings do not justify routine clinical screening for m.15312T>C and m.10114T>C alone. However, they support further investigation of genetic susceptibility to aminoglycoside-induced cochleotoxicity. Clinically, the high incidence of cochleotoxicity observed in this cohort reinforces the importance of structured ototoxicity monitoring, including ultra-high frequency audiometry where available, in patients exposed to aminoglycosides.

## Conclusion

This prospective cohort study found a high incidence of cochleotoxicity among South African MDR/RR-TB patients receiving kanamycin-based treatment. Significant threshold shifts were detected predominantly in the ultra-high frequency range before progressing into conventional frequencies over time. The mitochondrial variants m.15312T>C and m.10114T>C were observed only among participants who developed cochleotoxicity; however, no statistically significant associations were observed. These results should therefore be interpreted as exploratory and hypothesis-generating rather than confirmatory evidence of pathogenicity. Larger studies using broader genetic panels and adjustment for relevant clinical risk factors are needed to clarify the role of these and other mitochondrial variants in aminoglycoside-induced cochleotoxicity.
